# Can the EU be a federal democracy? Assessing the horizontal and vertical dimension of the EU government from comparative perspective

**DOI:** 10.1057/s41295-021-00265-2

**Published:** 2021-11-21

**Authors:** Jared Sonnicksen

**Affiliations:** Institute of Political Science, Technical University of Darmstadt, Dolivostr. 15, 64293 Darmstadt, Germany

**Keywords:** Democracy, European Union, Federalism, Government, Separation of powers

## Abstract

The European Union remains an ambivalent polity. This uncertainty complicates the assessment of its democratic and federal quality. Drawing on comparative federalism research can contribute not only to making sense of whether, or rather which kind of federalism the EU has developed. It can also enable addressing such a compounded, but necessary inquiry into the federal and democratic character of the EU and how to ascertain which *type* of democratic government for which *type* of federal union may be appropriate. The article first elaborates a framework to assess the dimensions of federal and democratic government, drawing on comparative federalism research to delineate basic types of federal democracy. Here the democratic dimension of government is taken as referring primarily to the *horizontal* division of powers (among ‘branches’) of government, the federal dimension to the *vertical* division of powers (among ‘levels’) of governments. The framework is applied to the government of the EU in order to gauge its own type(s) of division of power arrangements and the interlinkage between them. Finally, the discussion reflects on whether or rather how the EU could comprise a *federal democracy*, especially in light of recent crisis challenges and subsequent institutional developments in EU governance.

## Introduction

The European Union has experienced far-reaching integration in multiple policy areas, expanded scope of powers, and even the introduction of a Union citizenship. Yet much reticence remains to view the EU like a conventional political system in general and a federal one in particular. Varying, and in parts conflicting, definitions and preferences abound with regard to what the EU is and should be. Recent and current crises have only intensified political challenges to the EU, its member-state governments and citizenries: from the Eurocrisis and the ‘migration’ or rather ‘Schengen’ crisis, to the recent ‘Brexit’ and the ongoing Coronavirus/COVID-19 pandemic. In short, it seems that EU politics and policy challenges repeatedly trigger *polity* ones (see, for example, Lefkofridi and Schmitter 2015), confronting the EU again in the pending Conference on the Future of Europe.

The EU has long comprised an ambivalent, but also a ‘contested polity’ (Lord 2001). This complicates the management of numerous policy issues, but also the very evaluation of its democratic quality and, as the case may be, the proper approach to redress its deficit (see, for example, De Angelis 2020). Were one to still conceive the EU as international organization, albeit *sui-generis*, this may lead to particular conclusions: e.g. that either it is sufficiently democratic (as much, if not more than international organizations) or rather that its range of activity and powers have ‘gone too far’. However, matters are more complex. The EU has surely evolved into a unique arrangement of multilevel governance, but also taken on qualities of a system of *government* for one and of a *federal* system for another.

Federation emerged as one of the goals or leitmotifs of European integration from the outset.^1^ Nevertheless, European federalist visions have met with and still face much opposition. The widespread reluctance, and for many aversion, to using the notorious ‘f-word’ for the EU is reflected in recurring phases of treaty reforms and *finalité* debates, and not least in the midst of dealing with crises (Borriello and Crespy 2015; De Angelis 2020; cf. also Pollack 2005: 371ff.). Yet regardless of the normative or political (un)desirability of EU federalism, there is much reason to conclude, as others already have at earlier phases of European integration (e.g. Börzel and Hosli 2003; Sbragia 1993), that the EU amounts to a federal arrangement given its legal, structural and functional development. However, this inference alone does not answer *which kind of* federalism the EU has. Gaining more clarity on this question is warranted in order to comprehend the EU better (cf. Fossum and Jachtenfuchs 2017). It may also provide orientation for further analysis on EU reforms and democratization in particular. The crises of recent further underscore the relevance of these analytical questions.^2^

As recent scholarship in comparative federalism has pointed out (e.g. Benz 2009, 2020; Burgess and Gagnon 2010), the relationship between democracy and federalism in general cannot be taken for granted. The simple equation of ‘more federalism’ yielding ‘more democracy’ does not necessarily add up. For one, federal systems, like democratic governments, vary substantially. For another, democratic and federal elements or features can be combined or interlinked in one system in different ways that have implications for how democratic and federal government work. Comparative work on federalism and democracy and their interlinkage or *coupling* (see ibid.) can provide a fruitful approach to navigate a compounded, but necessary inquiry into the federal and democratic character of the EU and its system of government.

To this end, the following aims first to elaborate on the distinct dimensions of federal and democratic government, and basic types of their interlinkage or coupling in particular. The focus here is institutional and on the structural–functional organization of these two dimensions of government. The democratic dimension is taken here to refer primarily to the *horizontal* division of powers (among ‘branches’) of government, and the federal dimension chiefly to the *vertical* division of powers (among ‘levels’) of governments. Moreover, basic types of polities combining federalism and democracy are outlined. Thirdly, building on this analytical framework, the horizontal and vertical dimensions of government of the EU are then addressed in compact fashion. Admittedly, much of the complexity of the EU is reduced, though not oversimplified. The purpose of this endeavour is to capture the EU’s type(s) of democratic and federal arrangements and their combination to a pattern—or patterns, as the case may be—of federal democracy. Finally, drawing on the insights gained by taking this comparative federal–democratic perspective, the EU polity will be reflected with a view on whether or how it could better meet the conditions of a *federal democracy*.

## Federal and democratic government: distinct, but interlinked

There is a long-standing notion that federalism, with its separation of powers between levels of government, is inherently compatible with and even strengthens democratic government (for overview, see Burgess and Gagnon 2010; Gerring et al. 2005: 567ff; Levy 2007). It also appears to maintain considerable sway into the present. Indicative of this, as one noteworthy example, is Lijphart’s (2012) seminal work comparing democratic governments, and its ‘consensus model’ of democracy in particular, for which decentralized or federal government is among the definitive features (i.e. federalism is subsumed under the consensus-democratic type). In the context of the EU as well, federalism and ‘federalization’ have often been discussed as necessary prerequisite precisely for achieving democratization (see, for example, Trechsel 2005). However, without delving further theoretically, the world of actual government indicates that democracies need not be federal (the majority of democracies, including EU member states, are not), while federal systems are not necessarily democratic (Gibson 2013), nor do they inevitably enhance democracy. Particularly this point proves highly relevant to the kind of federalism developed in the EU (addressed further on). In short, the relationship between federalism and democracy is complex. This provides a conceptual point of departure for framing different types of federal democracies.

Rather than viewing them as part of one particular model of government, it is necessary first to conceive federalism and democracy as distinct dimensions of government. While both have separation of powers as core principle in common, each dimension involves *different* logics of dividing powers and of organizing structures and functions of government. Moreover, in principle and practice, federalism and democracy each have own variations in structural features and modes of operation relating to how powers are distributed: e.g. ranging from separated or shared; more towards autonomy and competition or rather cooperation, negotiation and consensus (Benz 2020; Burgess and Gagnon 2010; Hueglin and Fenna 2015). When combining the two regime dimensions into a federal democracy, the sure-fire result is a complex multidimensional polity, for better or worse. This becomes apparent with a closer view to these two different dimensions of distributing powers.

*Firstly*, in federalism, the division can be construed as principally *vertical* between *levels* of government: i.e. between the federal (or otherwise denoted superordinate) level of government and the governments of the constituent units. Unitary systems surely vary in terms of centralization or decentralization. However, federalism, according to the widely referenced concept by Elazar, is per se based on ‘non-centralization’ (Elazar 1985, 1987: 5). Consequently, irrespective of whether certain policy areas or processes are or become centralized or not, federal policies are necessarily distinguished by ‘a constitutionally structured dispersion of power’ (Watts 1998: 124), and thus an elemental diversity. The constituent units (e.g. cantons, provinces, or states) are aggregated in one polity, yet persist in their own right.

This constitutive distribution of powers between levels of government varies, in a most basic distinction, between *separating* and *sharing* powers. Keeping with the structural focus here, we may draw from comparative federalism research two ideal–typical models of *dual* and *cooperative* federalism, with the former more prone to *separation of* and the latter to *sharing* of powers (Hueglin and Fenna 2015: 136–141; Watts 2006). This dichotomy can pertain to areas of policy competence (e.g. defence, monetary, infrastructure, education, welfare etc.) as well as governmental functions (e.g. legislating, implementation, taxation, expenditures etc.). *Separation* is exemplified by dual federalism, where different levels are responsible for different policy areas within their own jurisdictions. Cooperative federalism on the other hand emphasizes *sharing*. This may entail constitutionally prescribed or otherwise institutionalized cross-level responsibilities for certain policy areas or rather a division of labour in the exercise of functions (e.g. one level primarily responsible for legislating and the other for implementing policy). The most intensive forms of cooperative federalism are cases of ‘joint decision-making’ (Scharpf 1988, 1997: 143ff.), with a cross-level interlocking between the governments at different levels whose consensus is required in order to reach decisions in those areas prescribing mandatory cooperation.^3^

*Secondly*, regarding democracy as dimension of government, the division of powers can be conceived as chiefly *horizontal* between *branches* of government. While authoritarian or other non-democratic systems can exhibit separated institutions with ‘checks and balances’ or even multiple ‘veto players’ (Tsebelis 2002), democratic governments and modern representative democracies in particular are fundamentally distinguished by free-and-fair popular elections. Maintaining again a structural–institutional focus, of particular concern then are the elected institutions of the executive and legislative branches. Accordingly, the organization of executive–legislative relations and their underlying division of powers pose the primary basis for differentiating *types* of democratic governments.

Here we may also draw from comparative research two ideal–typical forms of democratic government. Akin to federal types outlined above, we can distinguish them by their predisposition to *separate* or *share* powers between executive and legislative branches, namely *presidential* and *parliamentary* government, respectively (see, for example, Lijphart 1992; Samuels and Matthew 2010; Shugart and Carey 1992).^4^ Characteristic of the presidential type are separate elections of the chief executive and the legislature for fixed terms of office, while parliamentary systems in effect fuse the two in that the heads of government along with their cabinets are elected (formally or de facto) by parliament and depend on its confidence in order to govern (see ibid). As paramount distinguishing feature, the parliament in parliamentary governments, its majority specifically, can remove the (head of) government by vote of no confidence, whereas the legislature’s majority typically cannot depose chief executives in presidential systems (see ibid; also Huber 1996). Further typical features underscoring the predispositions towards separation or sharing of powers include, for example, the lack of powers to dissolve the legislature in presidential government, a prerogative typically afforded executives in parliamentary systems, in which compatibility between legislative mandate and executive office is usually (but not in all cases) permissible, but not in the presidential type.

As with the dual and cooperative types of federal government, these basic types of democratic government do not determine on their own policy outcomes. They do however matter for particular *patterns* of politics. The *horizontal* division of powers between executive and legislative—i.e. as rather strictly separated (presidential) or rather fused power-sharing (parliamentary)—coincides with how political decision-making and contestation unfold (cf., for example, Gerring et al. 2009; Samuels and Matthew 2010): e.g. whether along the lines of ‘government-versus-opposition’ dynamics and cohesive party discipline, typically parliamentary, or by variable cross-branch and cross-party coalitions on ad-hoc, case-by-case basis, typically presidential.

*Thirdly*, relations between branches and levels of governments surely differ according to the respective features of the horizontal and vertical dimensions of government. Building on these premises, we can conceptualize different types of institutional arrangements combining federal and democratic government, i.e. composite types of federal democracy. The basic types of federal and democratic features can be construed, again, as spanning from rather separation to rather sharing of powers. To delineate these types, Benz has proposed a concept of coupling, which refers to the type and degree of structural and functional interlinkages between institutions and arenas of democratic and federal politics (Benz 2009, 2020; see also Benz and Sonnicksen 2021). In the next step, I adopt this framework, with a focus on institutional, structural–functional features, i.e. on branches and levels of *government*.^5^

Two basic types of coupling between the federal and democratic dimensions of government may be summarized as *uncoupled* and *tightly coupled*, while a third rather mixed type is referred to as *loose coupling* (ibid). Firstly, *uncoupled* applies to arrangements of stricter separation of powers between executive and legislative branches of government as in a *presidential democracy* for one, and separation of powers *among* and with disjointed or few institutionalized interlinkages *between* levels of government as in *dual federalism* for another. The USA, a typical case in comparative politics both for presidential democracy and dual federalism, comes closest to the uncoupled ideal type. This is underlined further by bicameralism at federal level, where the second chamber, the Senate, consists of popularly elected senators: i.e. not delegated by, but rather ‘detached’ from state-level governments. Cooperation transpires even under such manifold separation of powers, but tensions arise especially when it comes to addressing cross-jurisdictional/cross-border problems. Deadlocks are prone to result, but can be resolved through consensus agreements or, failing that, then ‘escaped’ for instance by different levels of government resorting to unilateral actions within their own ambits.

*Tightly coupled* democratic and federal dimensions of government conversely implicates more intense interlinkage in multiple regards. This combination entails more sharing and even blending between executive and legislative, as in a *parliamentary democracy*, and is more cooperation and interdependence oriented, as in *cooperative* and especially *joint decision-making federalism*, with multiple institutionalized interlinkages between levels of government. The Federal Republic of Germany, as parliamentary democracy with a nearly unparalleled extent of joint decision-making, represents a prototypical case of *tightly coupled* federal democracy. This is also underlined by its Council model of a second chamber, the *Bundesrat*, in which members of state (*Land*) governments (i.e. the executive) are represented and co-decide on a large share of federal legislation. Here tensions also typically emerge in cases of cross-level cooperation. Representatives of different governments may voluntarily or be required to cooperate, but remain responsible to parliamentary majorities of their respective parliaments, so that the logic of competitive parliamentary government can ‘interfere’ with federal cooperation (Lehmbruch 2000; cf. comparing Australia and Canada, Sharman 1990). In contrast to the *uncoupled* variant, when cooperation and especially joint decision-making is required, failure to reach consensus results in deadlock at all levels, since a reversion to unilateral action *within* the levels of government is precluded.

The intermediary concept of *loose coupling* refers to processes of interaction in federal democracies that, structurally, may conform either to a tightly coupled or uncoupled type. Regarding division of powers, this variant is construed here as a combination of one dimension of government based on stricter separation and one more on sharing of powers. Loose coupling entails interaction and cooperation between governments but in flexible institutional arrangements, often underlined by voluntary coordination and opportunities for opt-outs. Canada provides a concrete example of a fused-powers system as (‘Westminster’) parliamentary democracy for one, and without much formal structural interlinkage between provinces and federal government for another, but with routinized intergovernmental relations and conferences for cooperative policy-making. Switzerland represents a separation-of-powers government (i.e. the parliament cannot depose the Executive Council, *Bundesrat*; there is no ‘government vs. opposition’ between the two branches), yet with an array of processes linking levels of government together for cross-level policy coordination and administration.

**Fig. 1:**
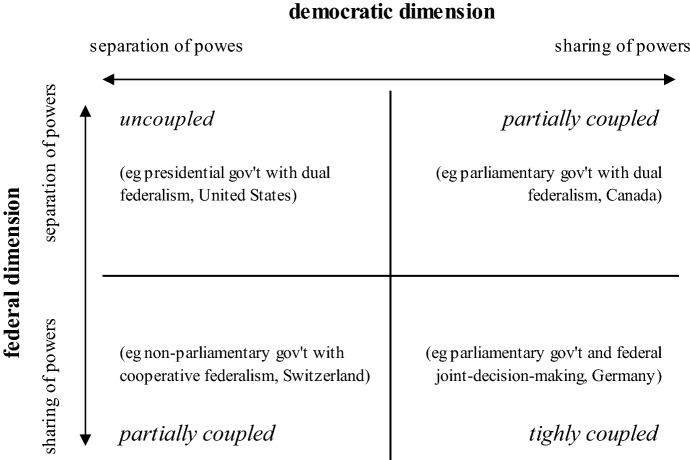
Combinations of democratic and federal government. Own revised depiction, adapted from the aforementioned illustrative cases (Canada, Germany, Switzerland, United States) elaborated further in Benz (2009, 2020).

While patterns of governing differ within types, individual cases, by policy area or over time, this framework captures several composite types of democracy and federalism (see Fig. 1). This framework not only provides a fruitful approach to comparing established federal democracies. Applying it to the EU also can contribute to determining more precisely what kind of system of multilevel, division-of-powers *government* has developed. Moreover, this may facilitate assessing normative implications of incongruences or ‘mismatches’, as when federal governance diverges from or exceeds the democratic government dimension and its capacity for commensurate legitimation.

## EU government in a multilevel system

The following revisits the EU polity based on the framework on types of federal democracies drawn above. Rather than chart the European Union in its entirety as political system (see comprehensively, e.g. Cini and Pérez-Solórzano 2019; Hix and Høyland 2011), the purpose is to capture the organization of branches and levels of government, with a specific view to determining, or approximating, its type of federal democracy. The horizontal dimension of government and the division of powers among the main governing EU institutions is briefly re-examined. In a second step, a condensed depiction of the vertical division of powers between levels in the EU is provided. Subsequently, a composite pattern of EU federal–democratic government is assessed, and critically engaged in light of more recent developments in European governance.

### Branches of government in the EU

The EU may lack *a* government in strict or conventional sense. However, the main institutions at EU level carry out many typical functions of government, and are embedded in a *horizontal* division of powers. This EU government encompasses ‘branches’ with separation of powers and ‘checks and balances’. In structural and functional terms, the Commission serves as an executive-type body, responsible for proposing legislation and monitoring subsequent implementation, and is *supranational* in character. The European Council, consisting of heads of state and government, likewise carries out executive functions, setting broader guidelines in particular, and is chiefly *intergovernmental* in character. The European Parliament (EP) and the Council of Ministers (Council) deliberate and decide on legislative acts, forming a legislative ‘branch’ which has developed functionally into a bicameral system (see, for example, Hagemann and Høyland 2010; Kreppel 2018). This applies especially to areas of ‘co-decision’ or the ‘ordinary’ legislative procedures (most notably, a vast share of areas directly related to regulating the Common Market).

The Council consists of members of national governments, enjoys decision-making competence in almost all EU governmental areas, but typically must reach broad consensus, and oftentimes unanimity. In instances of ‘co-decision’ with the EP, member-state representatives in the Council decide by qualified majority. The EP has broad legislative competences (co-decision is meanwhile the ordinary legislative procedure) and, unlike other EU institutions, direct democratic legitimation via popular elections. Parties in the EP have developed considerable inner-party congruence, with voting on personnel and bills following party lines, though in variable coalitions (see, for example, Hix and Høyland 2013). Hence, one can attest a strong bicameralism with a popular chamber (EP) and a member-state-based de facto ‘second chamber’ (Council) vis-à-vis an executive (Commission) that initiates proposals and ensures their implementation. To determine an EU type of government however, we need to consider further the relationship between executive and the legislative branches.

The EU has essentially two chief executives, one in the Commission and one in the European Council, each installed by different procedures and endowed with different responsibilities. The President of the European Council is appointed by consensus among the heads of member-state governments and exercises a predominantly coordinative role. Both the appointment of the Commission and its scope of responsibilities (Article 17 TEU) are more complex. To install a Commission President and a college of Commissioners (i.e. ‘cabinet’), (1) member-state governments in the European Council nominate the president and (2) national governments each nominate a further Commissioner, but both (1) and (2) are each subject to an EP vote of assent (see comprehensive, for example, Nasshoven 2010). The Treaty of Lisbon prescribed the European Council to take EP elections into consideration when nominating the Commission President (Article 17.7 TEU). For some, this seemed to pose a transition towards a parliamentary government. However, this shift has hardly come to fruition, which the EP elections and the European Council’s repeated disregard of the European political parties’ ‘Spitzenkandidaten’ underline further (Dawson 2019; Hobolt 2014). The relationship of EP and Council to the Commission has remained a non-parliamentary one for additional reasons.

**Fig. 2:**
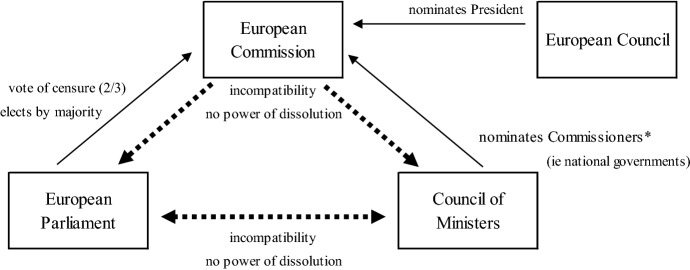
Separation of powers among EU institutions. *Source*: own depiction

Unlike parliamentary systems, the EP has no effective vote of confidence, but rather a censure vote requiring a two-thirds majority and limited in its application (Article 234 TFEU). Conversely the Commission lacks competences to discipline either the EP or the Council with votes of confidence or to dissolve either ‘chamber’. Further underlining stricter separation of powers in EU government is incompatibility, i.e. unlike most parliamentary systems, members of the EP and of the Council (member-state governments) cannot simultaneously be part of the Commission. Unsurprisingly, governing in the EU does not conform to politics along the lines of ‘government versus opposition’, not even within the EP and even less so among EU institutions (Fig. 2).

EU government entails mutual *checks and balances* in legislative processes as well as in the nomination and confirmation of chief executives. The EU institutions depend on one another to govern. However, they maintain stricter separation of powers given membership incompatibility between institutions, lack of executive powers to dissolve legislative institutions, and lack of legislature power to remove executives by regular-majority vote of no confidence. From this follows that, in the horizontal dimension, the EU government resembles a non-parliamentary, rather *presidential* type governmental arrangement on the one hand (cf. also Fabbrini 2004; Kreppel 2011; Sonnicksen 2017), but bereft of the typically coinciding democratic linkage on the other, a popularly elected chief executive.

### Levels of government in the EU

Concerning the *vertical dimension*, the EU is widely accepted as a paradigmatic case of multilevel governance (see, for example, Hooghe and Marks 2001; Piattoni 2010). The EU treaties refrain from referring to federalism per se, nor do they include explicit references to sovereignty. However, they do exhibit formal indications of not just a multilevel, but even a federal arrangement: for instance, a ‘reservation’ of powers to the member states (e.g. Article 4 TEU, reserving competences not conferred upon the Union to the Member States) in combination with principles of ‘subsidiarity’ and vertical distribution of powers (e.g. Article 5 TEU, by which Union competences ‘are governed by the principle of conferral’). The treaties allocate various political and institutional responsibilities for instance by policy area. One may summarize three basic types or ‘patterns’ of competence allocation (and with that, ‘governance modes’), namely supranational, intergovernmental and joint competences (see already Scharpf 1994; cf. also comprehensive Héretier and Rhodes 2010). In supranational areas, i.e. falling exclusively under the ambit of the Union such as trade, competition and, for ‘Eurozone’ members, currency policy, the responsibility for implementation lies predominantly with Union-level institutions (e.g. the Commission, in some cases the European Central Bank, etc.). In more intergovernmental areas like foreign and security policy, the European Council, Council and the member states are primarily responsible for making decisions—typically by unanimity or broad consensus—and carrying them out. The third main variant refers to areas of joint responsibility between the Union and the member states (see Articles 4, 5 and 6 TFEU), representing by now the largest share of EU policy.

**Fig. 3:**
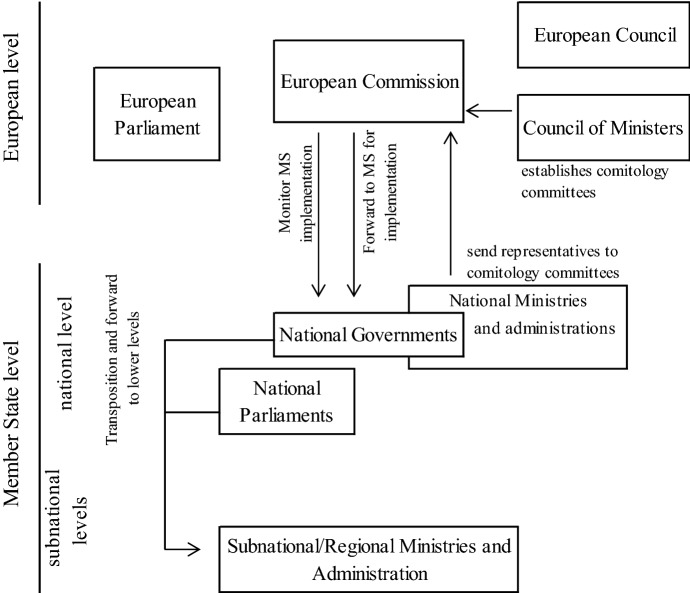
The multilevel executive and implementation (ordinary legislation). *Source*: own depiction

In areas of joint responsibility especially, the treaties set forth that member states ‘shall adopt all measures of national law necessary to implement legally binding Union acts’ (Article 291.1 TFEU), i.e. implementation at the member-state level. Moreover, there is a far-reaching multilevel interlinkage of the executive reflected in the rules on administrative cooperation (Article 197 TFEU-L). This means in principal that member-state actors—not only from governments, but also national and subnational administrations—are decisive in implementing EU law. This responsibility for implementation comes in addition to the weighty position of *member-state governments* in EU legislative matters through their incorporation in a de facto ‘second chamber’, the Council. The duty, in turn, to enforce these processes falls upon the Commission, which ensures the ‘application of the treaties’ (Article 17 TEU) and takes care that Union policies are implemented (see also Fig. 3). Several control and monitoring instruments are at the disposal of the Commission (Article 17.1 TEU; Articles 105, 258 and 259 TFEU), such as the competence to start infringement procedures against non-compliant member states or bring them before the European Court of Justice.

The Commission and multiple administrative and regulatory agencies linked to it in various ways have developed into a wide-ranging European bureaucracy or *Eurocracy* (Kelemen and Tarrant 2011). Further European agencies and offices have been established for coordinating and regulating individual policy areas, with variable degrees of (in)dependence and discretion, being loosely or tightly bound to the Commission, other EU institutions and/or the member states (see, for example, Egeberg and Trondal 2017). These institutional and procedural features also point to development of a multilevel administration and *government*.

With the transition from Community to wider European Union, it has already been concluded that a ‘regulatory federal’ (Keleman 2000) and even ‘executive federal’ (Schütze 2010) system has developed. Surmising moreover from the concise sketch above, the EU *vertical* division-of-powers dimension encompasses multiple elements of federalism—even if not nominally so. These features range from dualistic separation-of-powers federalism (e.g. conferral of powers to the Union, reservation of other powers to the member states), an extensive scope of cooperative federalism (e.g. numerous areas prescribing intergovernmental cooperation and coordination), and not least the *joint* decision and administration of European legal acts. The EU level may lack an own *Kompetenz-Kompetenz*, in the sense that the EU ‘government’ could alter its own scope of powers (see, for example, Beck 2011). However, national governments in federal systems normally also lack the power to unilaterally alter the constitutional distribution of powers.

### EU polity: a pattern of mixed federal and limited democratic government

The EU comprises a multilevel system with extensive, though variable vertical separation and sharing of powers. Along with the Commission, numerous actors from multiple levels and institutions of government are included into the process of implementing European legal acts, participating, controlling, influencing and shaping implementation at multiple stages and places. The EU executive can be divided structurally on a horizontal level into a rather supranational part, anchored mainly in the Commission, and an intergovernmental, member-state oriented part, institutionalized most saliently in the Council and the European Council. The combination of this complexity with variability between supranational, intergovernmental, and community modes of governance in the EU multilevel system allows for its conceptualization *in toto* as a ‘loosely coupled’ (Benz 2010) federative arrangement. However, governing in the EU far exceeds loose coupling in the passage and implementation of Union legal acts, especially those reached by co-decision, comprising together a system of *joint decision-making*.

**Fig. 4:**
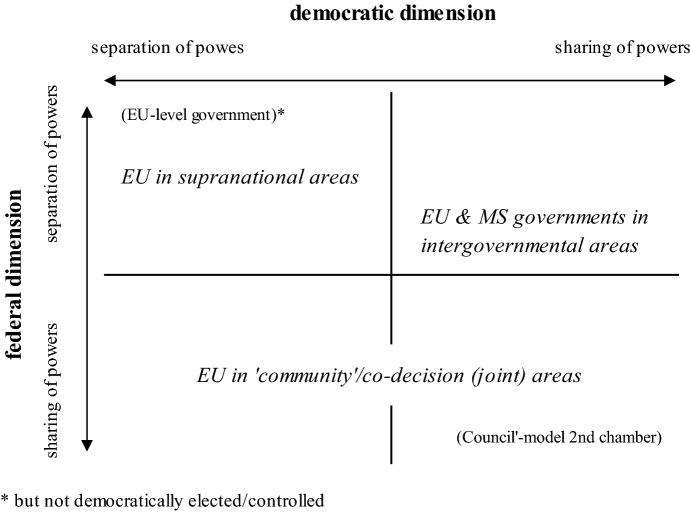
Pattern(s) of EU government between separation and sharing of powers. *Source*: own depiction

On the one hand, regarding the *horizontal* dimension, the EU *government* corresponds with a separation-of-powers type system. It is structurally and functionally akin to presidential systems of separation between executive and legislative, though unlike, for example, the US case, without popular elections of the EU executive or second chamber. The EU *vertical* dimension of government, on the other hand, especially where joint decision-making and administration are concerned, resembles rather the structures and functions of Germany’s tightly coupled cooperative federalism (cf. also Kreppel 2011), but without an EU-level government responsible to the EP as popularly elected first chamber. The interlinkage between member-state governments co-deciding EU legislative matters that they subsequently are responsible for implementing at national level reveals remarkable similarities to the German ‘Bundesrat’ model. However, the EU differs from this case of federal democracy in that, at EU level, the system of government diverges categorically from parliamentary democracy: i.e. no confidence relationship between legislature and EU chief executives. Indeed, the EU departs from federal democracies in lacking a democratically elected and accountable government altogether (Fig. 4).

Thus we arrive at a composite picture of the horizontal and vertical organization of branches and levels of government in the EU. While the EU bears striking similarities to other federal systems in various regards, it also exhibits divergences or ‘gaps’ between its federal governance and representative–democratic government. Gauging by established federal democracies, several commensurate democratization reforms could span from, for instance: (1) bringing the EU in line with a parliamentary–democratic federalism by which the Commission becomes a government responsible to *and removable* by an EP majority (e.g. akin to the German tightly coupled model); (2) the direct election of the Commission President in line with a presidential-democratic federal order (e.g. akin to the US uncoupled model, at least at EU level); or (3) maintaining a non-parliamentary, separation-of-powers EU-level government ‘as is’ and rather pursuing democratization by introducing facultative referendums, thus embedding EU governance in the ‘shadow’ of potential plebiscites (e.g. akin to the Swiss ‘loosely coupled’ model). The already-established federalism of the EU warrants consideration of such democratization reforms. But further complexity emerges, for one, given the larger diversity of governance modes in the EU than captured above. For another, changing patterns of governing in the EU raise additional challenges to realizing a more democratic federalism.

### Changing patterns of governing as federal–democratic challenges

The prior analysis affirms that the community method of governing in the EU (i.e. ordinary legislative procedure with joint decision-making and cooperative-federal implementation) raises concerns of a democratic deficit which has not been resolved. The intergovernmental mode of governing, in contrast, would appear much less invasive on the autonomy of member states and thus less demanding of a ‘Europeanized’ democratic legitimation. Common decisions in intergovernmentally constituted areas have typically required unanimity and, when barring this, further integration could be reached at least though constructive abstentions and/or the permission of opt-outs. This sort of loosely coupled arrangement has also been likened to modes of ‘treaty federalism’ in the Canadian case (Hueglin 2013; cf. also Verdun 2016). More reliance on this approach to governing could allow for variable, ‘differentiated integration’ (Fossum 2015; Schmidt 2019): accordingly national governments could reach voluntary agreements, while maintaining substantial latitude for achieving common goals and without hierarchic enforcement of compliance by the supranational level.

What has complicated, indeed exacerbated, matters in recent years however, has been precisely the shift to the mode of a ‘new intergovernmentalism’ (Bickerton et al. 2015). This pattern has intensified most notably in the wake of the currency, sovereign debt and wider financial European crises, followed by a so-called ‘migration crisis’ induced by a flux of migration—the EU crisis lying rather with its breakdown in cross-European coordination. The democratic deficit already attributed to ordinary patterns of governing within the Common Market becomes particularly problematic in the intergovernmental mode when it turns coercive. This applies especially for the creation of de novo bodies (e.g. EFSF and ESM) to implement conditionality or austerity policy on fiscally beleaguered member states; moreover, these developments have transpired under the conditions of a persistent lack of a common EU welfare regime as corrective counterpart to integration that one-sidedly favours the market freedoms of people, goods, capital and services (see, for example, Matthijs 2017; Scharpf 2015). In the case of the Eurocrisis, the fiscal and economic governance responses were not led by *the* EU government, but rather national governments. Further austerity and budget consolidation policies determined by the Councils and the strengthening of the Commission’s role in monitoring national compliance herewith, have intensified ‘executive federalism’ of the EU, but without any parallel extensions of democratization (Crum 2013; Fabbrini 2016). This rather propels a further decline in accountability of European governance to national parliaments or the European Parliament. The failure of national governments to manage common approaches like in the ‘migration crisis’ or current coronavirus pandemic could have severe implications for democratic legitimacy and support for the EU, the ‘integration project’ and even national governments.

From democratic–federal perspective, a resort to more intergovernmentalism under current conditions in pursuit of further integration can hardly provide a viably legitimate approach. Certainly the world of democratic federalism does not provide any comparable model or practice. One would search in vain for a democratic federation that has no polity-wide democratically legitimated government, or where subnational governments take over leadership of the polity via intergovernmental conferences. That is, unless one looked at looser confederal models of the past, which were also severely more limited in scope and trajectory of powers than is the case in the current EU (cf. Glencross 2009). If national governments continue to assume leadership at European level, they would, by any minimal representative-democratic standard, have to take on corresponding responsibility. Under given conditions though, member-state governments are responsible to their national parliaments and electorates. The latter in turn are structurally and institutionally wholly detached or *uncoupled* from one another (i.e. citizens do not vote for parliaments of other member states). As a result, and in absence of an EU-level government to elect and, as the case may, reward or sanction, then *national* elections would also have to serve as channel for contestation over *European*-level politics and political (non-)decisions.

Compared with federal democracies, the federalism developed in the EU does not adequately conform to representative–democratic governments. The crises management of recent years has even exacerbated this deficit. Crises may generally tend to be the ‘hour of the executive’ in any polity. However, the EU polity lacks an overarching democratic government that may allow for tolerating, not to mention legitimating a temporary ‘stretch’ of the EU executive, be it the Commission or the Council. At the bottom line, the analysis of how the EU system of government has evolved and recent developments at latest reveal that, to be a federal democracy, a democratization of European federalism appears all the more urgent.

## Conclusion

The Treaty of Lisbon, the last large-scale revision of the European Treaties, prescribed to enhance multiple democratically and federally relevant principles. However, the dynamics of EU governance have failed to bring about, for instance, either the transition to EU parliamentary government or an effective extension of national parliaments (see, for example, Bevir and Phillips 2017). Concerning representative democracy, the requirement of member-state governments to consider EP elections in selecting the Commission President was celebrated as step towards a parliamentary model. While this would constitute a stark shift away from the EU government’s separation of powers, it also does not appear as a realistic prospect. The member-state governments enjoy a preeminent position at EU level, and the current Commission installed after the EP elections of 2019 confirmed national governments’ willingness *and ability* to discard European Political Parties’ ‘Spitzenkandidaten’ without ramification (Dawson 2019; Heidbreder and Schade 2019). The Treaty likewise prescribed a commitment to enhancing the position of national parliaments in the EU. However, the concrete inclusion of an Early Warning Mechanism has led neither to the fruition of a ‘virtual third chamber’ (Cooper 2012), nor the expansion of horizontal interparliamentary cooperation that could counteract the long-attested executive dominance in the EU (see, for example, Bellamy and Kröger 2016). On the contrary, the executive dominance grew not just intensely but also asymmetrically among member states in crises of recent. The result is a rather sobering balance on the EU as a federal democracy.

The comparative federal–democratic framework applied here demonstrates much analytical merit. It has helped to capture the constitution of the horizontal and vertical division of powers in the EU, but also of what *kind* or type in each dimension. While not a *democratic* government, the EU institutions make up a system of separation of powers among its branches. At the same time, the assessment of the vertical dimension does not just reaffirm that the EU is sui-generis yet ‘somehow federal’. Of course, the EU remains one of a kind. But comparison reveals that the EU multilevel system corresponds to a large extent to one of legislative and administrative cooperative federalism with joint decision-making. The approach taken here thus allows for identifying more specifically the EU character in comparison with federal democracies: its—hitherto unparalleled—combination of stricter separation of powers at the level of (supra)national government with an arrangement of vertical interlocking and extensive joint decision-making between levels of government. Leaving aside such questions as to whether the EU has or even could have a *demos* or at most *demoi* (cf., for example, Nicolaïdis 2013; Risse 2014; Ronzoni 2017), it remains difficult but by no means impossible to assess a commensurate democratization approach.

Complicating matters though is that much of multilevel governance in the EU still falls outside of the area of community or joint tasks and respective decision-making modes, and are more intergovernmental in character. Where intergovernmentalism has tended to predominate, the question of EU-level democracy could long be deemed less pressing, as autonomy of member states and their democratically elected governments enjoyed safeguards, e.g. through unanimity rules, leverage for opt-outs and divergences, or resort to voluntary cooperation. On the other hand, intergovernmental governance in the EU has witnessed a shift in recent years to executive dominance, no less asymmetrically wielded among the member-state governments. Under such conditions, the EU multilevel government not only continues to have a demos- but even *demoi*-constraining effect.

Consequently, whether one subscribes to the concept of the EU as a mixed Union of people and states, one of a compound republic, or rather a confederal association of states where only the co-existence of *peoples* are possible, the comparative federal–democratic framework adopted here allows, I submit, for us to draw a clearer conclusion: federalism and democracy are out of balance in the EU. For not only has the extent of democratization of Union government *not* coincided with the extension of the scope and trajectory of supranational and joint decision EU governance so far. Intergovernmental governance *would also* appear to exceed its basis of member-state grounded democratic legitimacy. This reveals a challenge to democratizing an EU government as it stands.

To comprise thus a *federal democracy*, while many options are conceivable, in short, two *basic federal–democratic* routes to this end can be summarized. One route implicates specifically federal measures towards *uncoupling* and returning competences to the member states where institutions and processes of *democratic* government—not just complex separation of powers—still reside in Europe. Such step would apply to supranational and ‘communitarized’ areas, but also the intergovernmental arena where formerly loosely coupled governance has taken on a peculiar pattern of a coercive and asymmetric one. The alternative route European governments could take lies in pursuing a fundamental democratization of the EU ‘federal government’, including Europeanization of EP elections *and* of popular control over the EU executive, whether, for example, by its popular election or its more consistent transition towards a parliamentary government elected by and responsible to the EP majority that would likewise have more co-determination in areas that are organized intergovernmentally. Otherwise, the EU and its member states risk remaining stuck to meddling through a system of *federalism* many still refuse to admit has already been achieved, while failing to fulfil standards of *democracy* the Treaties, EU institutions and member-state governments claim to be committed to.

## Footnotes

1 See the Schuman Declaration (1950), e.g.: “The pooling of coal and steel production should immediately provide for the setting up of common foundations for economic development as a first step in the *federation of Europe*”, (emphasis added).

2 For instance, the Conference on the Future of Europe has again placed reforms on the EU political agenda, while after Brexit, the EU has lost a conventionally rather adamant opponent of the ‘F-word’ in Europe, which could (though not necessarily) facilitate the reform debate in this sense.

3 Of course varieties and complexities of federal systems have been reduced here. The joint decision-making concept—see Scharpf et al. (1976)—is important case in point, which differentiated between systems of compulsory and voluntary cooperation among governments, among other things, while more recent research has elaborated even further varieties of joint decision-making; see esp. Benz et al. (2016). However, for sake of brevity and concise conceptualization, the analytical framework here foregoes an in-depth elaboration of the sub-variants of basic types of federal systems.

4 There is much discussion in the relevant comparative government literature on demarcations between presidential and parliamentary government and the ‘hybrid’ case of semi-presidential systems (for overview, see e.g. Fruhstorfer 2016). While the office of head of state is elected popularly, the head of government remains responsible to the parliament and its majority in particular. Consequently, I view ‘semi-presidential’ systems here as a subtype of parliamentary system.

5 The analytical framework here leaves out other—admittedly highly relevant—features such as intergovernmental relations or, the federal character of the party system, among others, to focus on *government*.
